# Correction: Removal of iodine from organic media using diethylene triamine-grafted vinylbenzyl chloride-divinylbenzene resin

**DOI:** 10.1039/d6ra90061e

**Published:** 2026-07-06

**Authors:** Aimon Masood, Kashmala Khaliq, Adil Khan, Munib Ahmed Shafiq, Samreen Shehzadi, Iqra Rafique, Ramzan Akhtar, Saeed Omer, Mohsin Ali Raza Anjum, Sajid Iqbal, Muhammad Saifullah

**Affiliations:** a Department of Chemistry, Pakistan Institute of Engineering and Applied Sciences (PIEAS) Islamabad Pakistan; b Department of Chemistry, University of Poonch Rawalakot AJK Pakistan; c Chemistry Division, Pakistan Institute of Nuclear Science and Technology (PINSTECH) P.O. Box 45650, Nilore Islamabad Pakistan saifi.551@gmail.com; d Central Analytical Facility Division (CAFD), Pakistan Institute of Nuclear Science and Technology (PINSTECH) P. O. Box 45650, Nilore Islamabad Pakistan; e Department of Chemistry, Government College University Faisalabad (GCUF) Faisalabad Pakistan; f Department of Nuclear and Quantum Engineering, KAIST 291 Deahak-ro Yuseong-gu Daejeon 34141 Republic of Korea sajid1@kaist.ac.kr

## Abstract

Correction for ‘Removal of iodine from organic media using diethylene triamine-grafted vinylbenzyl chloride-divinylbenzene resin’ by Aimon Masood *et al.*, *RSC Adv.*, 2026, **16**, 27611–27620, https://doi.org/10.1039/D6RA01669C.

The authors regret that an unintended image duplication occurred in Fig. 1a of this manuscript. Included below is the revised figure where the correct image is presented for Fig. 1a.

**Fig. 1:**
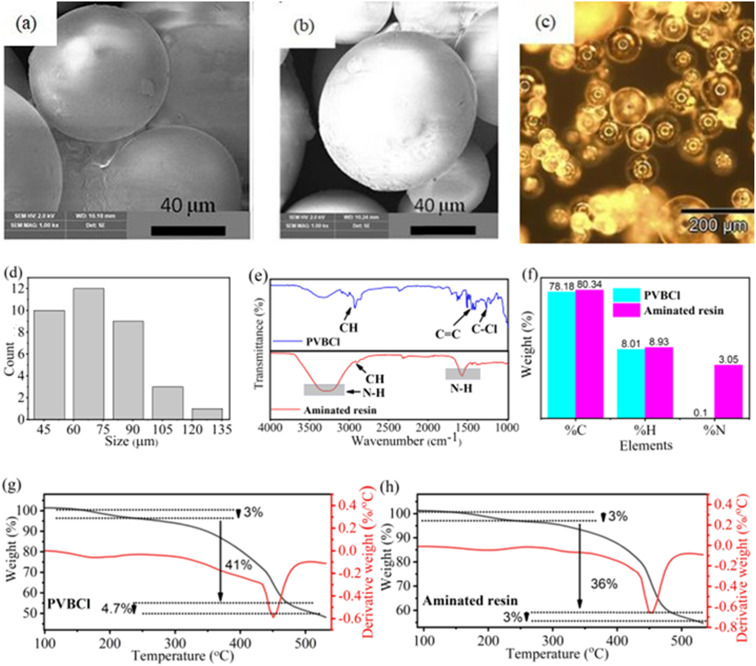
SEM images of (a) PVBCl pristine resin (before functionalization), (b) aminated resin (after functionalization), (c) optical image of PVBCl resin beads, (d) histogram of bead's size distribution (PVBCl), (e) FTIR spectra of the PVBCl pristine and aminated resins, (f) organic elemental analysis of PVBCl pristine and aminated resins, (g) TGA-DTG profiles of PVBCl pristine, and (h) TGA-DTG profiles of aminated resin.

The Royal Society of Chemistry apologises for these errors and any consequent inconvenience to authors and readers.

